# Telotristat ethyl affects tumour‐fibroblast crosstalk in small intestinal neuroendocrine tumours

**DOI:** 10.1111/jne.70094

**Published:** 2025-09-25

**Authors:** Harry Hodgetts, Maria Castanho Martins, Luohai Chen, Andrew R. Hall, Tu Vinh Luong, Dalvinder Mandair, Martyn Caplin, Krista Rombouts

**Affiliations:** ^1^ Regenerative Medicine and Fibrosis Group, Institute for Liver and Digestive Health Royal Free Hospital, University College London London UK; ^2^ Neuroendocrine Tumour Unit, ENETS Centre of Excellence Royal Free London NHS Foundation Trust London UK; ^3^ Sheila Sherlock Liver Centre Royal Free London NHS Foundation Trust London UK; ^4^ Cellular Pathology Department Royal Free London NHS Foundation Trust London UK

**Keywords:** fibroblast, GOT1, neuroendocrine tumour, small intestine, telotristat ethyl

## Abstract

Small intestinal neuroendocrine tumours (SI‐NETs) are associated with mesenteric fibrosis, which causes significant morbidity and mortality. Telotristat ethyl was developed to treat carcinoid syndrome in SI‐NET patients. Recent studies indicated telotristat ethyl could have anti‐tumour activity; however, the mechanism remains unclear. This study aimed to investigate the effects of telotristat ethyl on SI‐NET–fibroblast crosstalk in tumour progression and mesenteric fibrosis. A co‐culture paracrine model with GOT1 (tumour) cells and LX2 (stromal) cells was optimized. Cells were treated with conditioned medium with/without telotristat ethyl followed by RNA sequencing and Gene Set Enrichment Analysis. Quantitative RT‐PCR, immunohistochemistry, and Western blot were performed on first and second tier targets in tissue from 34 SI‐NET patients grouped into categories of mesenteric fibrosis severity. Telotristat ethyl significantly decreased proliferation and serotonin secretion in a dose‐dependent manner in GOT1 cells. GSEA data indicated ECM‐related reactomes were downregulated in GOT1 cells grown in conditioned medium of LX2 cells with telotristat ethyl. *LAMA5*, *COL6A2*, and *COL12A1* expression was significantly increased in mild and severely fibrotic patients. Immunohistochemistry determined the localization of proteins such as COL4A2 in the stroma and ADAM12 in tumour cells. Protein analysis of second tier targets showed differences in expression, including β‐catenin, which was significantly upregulated, and pAKT/AKT, which tended to increase in primary tumour compared to normal SI. Telotristat ethyl affects the expression of genes associated with the ECM and interferes with SI‐NET–fibroblast crosstalk. Further analysis is required; however, this study represents an important step in understanding the mechanisms of telotristat ethyl when treating SI‐NET patients.

## 1 INTRODUCTION

The small intestine is one of the most common sites for neuroendocrine tumours (NETs), and the incidence rate has increased in recent decades.^1^, ^2^ Small intestinal NETs (SI‐NETs) are often associated with elevated levels of serotonin, leading to the development of carcinoid syndrome.^3^ Carcinoid syndrome is a collection of symptoms, including flushing, abdominal pain, and diarrhoea, which impacts patient quality of life and can lead to life‐threatening complications.^4^ The main treatment for carcinoid syndrome over the past few decades involves the use of somatostatin analogues; however, many patients become resistant to these drugs and can develop refractory carcinoid syndrome.^5^ As such, a new method of carcinoid syndrome treatment involving the inhibition of serotonin synthesis has been explored.

Tryptophan hydroxylase (TPH1) is an enzyme involved in the rate‐limiting step of serotonin synthesis, and telotristat ethyl (TE) was developed as a molecule to inhibit this enzyme for the treatment of carcinoid syndrome diarrhoea.^6^ Subsequent clinical trials have validated the efficacy of TE in decreasing circulating serotonin (indicated by decreased 5‐hydroxyindoleacetic acid, 5‐HIAA, in patient urine) and controlling carcinoid syndrome symptoms.^7^ As such, TE is being increasingly prescribed for SI‐NET patients with carcinoid syndrome.^8^

Recent studies have also indicated TE's potential for anti‐tumour activity in SI‐NET patients. One study, which assessed 200 patients, showed that treatment with TE for at least 6 months stabilized SI‐NETs and induced tumour shrinkage, with an average reduction in tumour size of 0.59 cm.^9^ A follow‐up study also highlighted improvement in progression‐free survival and clinical outcomes.^10^ Despite these studies, the underlying mechanism of the anti‐tumour effect of TE remains unsolved.

Previous studies showed that serotonin itself might act as an autocrine factor favouring lung and SI‐NET cell line proliferation and survival (^11^); however, other recent studies also suggest that the inhibition of serotonin had no effect on pancreatic NET cell lines in vitro.^12^ This could indicate that the anti‐proliferative effects of TE could derive from the crosstalk between the tumour cells and the tumour microenvironment. These conflicting results also highlight the need for further research. The potential role of TE in the interactions of tumour cells and stromal cells in SI‐NETs has hardly been studied. Given the increasing evidence indicating the pivotal role of cancer‐associated fibroblasts in cancer proliferation and invasion,^13^ this could be a particular area of interest in elucidating the mechanism of TE on NET cells.^14^

Therefore, this study aimed to investigate the effects of TE on SI‐NETs and fibroblasts and further delineate the underlying mechanism of TE in the crosstalk between SI‐NETs and fibroblasts.

## 2 METHODS

### 2.1 Cell lines, culture and treatment

GOT1 cells, a midgut NET cell line derived from a human liver metastasis, were a gift from Ola Nilsson, Sahlgrenska Cancer Center, University of Gothenburg, Sweden.^15^, ^16^ LX2 cells, a well‐characterized cell line obtained from human hepatic stellate cells (hepatic fibroblast cells), were used as the stromal cell line. LX2 cells were kindly gifted by Professor Scott Friedman, Mount Sinai, NY, USA.^17^ Both cell types were cultured in RPMI 1640 medium (Gibco) supplemented with 10% foetal bovine serum (FBS; Gibco), 1% penicillin/streptomycin (Gibco), insulin (Sigma‐Aldrich), transferrin (Sigma Aldrich), and L‐glutamine (Gibco). Both GOT1 and LX2 cells were cultured in T175 flasks (GOT1: Thermo Scientific; LX2: Cellstar) in an incubator at 37°C and 5% CO_2_. Cells were treated with Telotristat etiprate (salt form) active ingredient (Purity >95%) and were dissolved in dimethyl sulfoxide (DMSO), and all the experiments described in this article were performed with Telotristat Ethyl and referred to in the text as TE.

### 2.2 Paracrine in vitro model of GOT1 and LX2 cells

Following optimisation experiments for the concentrations of TE and FBS to be used (Figure S1), GOT1 cells were cultured in T75 flasks at a density of 1 × 10^7^ cells/flask, and LX2 cells were cultured in T175 flasks at a density of 1 × 10^6^ cells/flask. After 48 h of incubation in complete culture medium with 10% FBS, supernatants were discarded, and cells were washed three times using HBSS (Gibco). 10 mL (GOT1) or 20 mL (LX2) of medium with 0.5% FBS, supplemented with or without 10^−6^ mol/L of TE, was added. Cells were incubated for a further 72 h, and the supernatants were collected and centrifuged at 1200 rpm for 10 min. Supernatants were collected and stored at −80°C until further usage.

Next, GOT1 and LX2 cells were cultured in complete medium in 6‐well plates at a density of 2 × 10^5^ cells/well and 2 × 10^4^ cells/well, respectively. After 48 h, supernatants were discarded, and cells were washed three times with HBSS. GOT1 cells were treated for 72 h with (1) 2 mL medium with 10% FBS (complete medium, CM), (2) medium with 0.5% FBS (0 M), (3) conditioned medium from LX2 cells (CdML2), (4) conditioned medium from LX2 cells which were also treated with 10–6 mol/L of TE (CdML2T), (5) conditioned medium from LX2 cells supplemented with 10–6 mol/L of TE (CdML2 + Telo). Similarly, LX2 cells were treated for 72 h with (1) 2 mL medium with 10% FBS (complete medium, CM), (2) medium with 0.5% FBS (0 M), (3) conditioned medium from GOT1 cells (CdMG1), (4) conditioned medium from GOT1 cells which were treated with 10–6 mol/L of TE (CdMG1), 5) conditioned medium from GOT1 cells supplemented with 10–6 mol/L of TE (CdMG1 + Telo).

### 2.3 RT2 profiler PCR array analysis

RNA extraction was performed using the Promega ReliaPrep™ RNA Miniprep System, and concentration was measured using a Nanodrop 1000. RNA samples from three biological replicates of each condition from each cell line were used as biological samples for further analysis. 200 ng of each RNA sample was used for cDNA synthesis by using the RT2 First Strand Kit (Qiagen). The Human Cancer Pathway Finder array (Qiagen) was used to assess changes in gene expression in GOT1 cells, and the Human Fibrosis array (Qiagen) was used for analysing gene expression changes in LX2 cells.

### 2.4 RNA sequencing

RNA sequencing was performed in collaboration with UCL Genomics and was conducted using three RNA biological samples per condition for each cell line under investigation, as we have described previously.^18^ Briefly, the library preparation of 100 ng total RNA was processed using the NEBNext RNA Ultra II kit with Poly A+ selection (p/n E7760 & E7490) according to the manufacturer's instructions. For sequencing, the libraries to be multiplexed in the same run were pooled in equimolar quantities calculated from Qubit and Bioanalyser fragment analysis. Samples were sequenced on the NextSeq 500 instrument (Illumina, San Diego, US) using a 75 bp single read run with a corresponding 8 bp unique molecular identifier (UMI) read.^18^

### 2.5 Gene set enrichment analysis (GSEA)

GSEA was performed in collaboration with UCL Pathogen Genomics Unit (PGU). All gene lists used were retrieved from MSigDB version 7.2. The following custom set includes: C2 Reactome (complete), C6 Oncogenic (complete), C8 Cell type (only liver) and any gene set with a name matching ‘neuroendocrine.’ Only gene sets containing between 15 and 500 genes were used for analysis in accordance with GSEA default parameters. The number of permutations was increased to 10,000 to increase statistical robustness. Five different conditions for each culture were analysed, with the GSEA producing 20 different comparisons. A leading‐edge analysis was also performed to show the overlapping genes between different reactomes.

### 2.6 Gene expression by RT‐qPCR

Genes selected from the GSEA analysis were further analysed on human SI‐NET patient tissue. A cohort of patients was grouped into 4 distinct categories with graded severity of mesenteric fibrosis: patients with no mesenteric metastases (all patients with a mesenteric metastasis had some evidence of fibrosis) (*n* = 3), minimal (*n* = 6), mild (*n* = 14), and severe fibrosis (*n* = 11) as previously described.^18^ The demographic and clinical characteristics of patients and the multidimensional assessment of mesenteric fibrosis used to classify patients into distinct groups of mesenteric fibrosis severity can be found in Supporting Information S1. Briefly, snap frozen human tissue samples of normal small intestine, primary tumour, and mesenteric tumours were homogenised using a TissueLyser II (Qiagen), and RNA was extracted using the RNeasy Mini Kit (Qiagen). RNA concentration and purity were determined using a NanoDrop 2000 spectrophotometer. 500 ng total RNA was reversed transcribed using the High‐Capacity cDNA Reverse Transcription Kit (Thermo Fisher Scientific).^18^ The PCR reaction was performed using the Applied Biosystems 7500 Fast Real Time PCR machine with TaqMan™ Fast Advanced Master Mix (Thermo Fisher) and Assays‐on‐Demand (Thermo Fisher; Supporting Information Table 1). Gene expression was normalised using GAPDH, and the fold changes were calculated relative to normal small intestine as previously described.^18^

### 2.7 Immunohistochemistry

The localization of proteins such as CDH1, P4HB, TIMP1, COLA4, ADAM12, and TMPRSS6 was assessed. Sections of FFPE tissue from normal small intestine, primary, and mesenteric tumours from patients in each group of fibrosis severity were used (*n* = 3). Sections were de‐paraffinized and hydrated through serial xylenes (3 × 5 min) and industrial denatured alcohol (IDA) (3 × 2 min, 100%, 100%, 90%). Antigen retrieval protocols for each target are detailed in Supporting Information Table 2. Slides were soaked in TBS with 0.04% Tween‐20 (Sigma) for 5 min, then in Bloxall solution (Vector) for 10 min. The slides were washed in TBS for 5 min and blocked in 2.5% normal horse serum (Vector) for 15 min, and then incubated for 1 h with primary antibodies. Binding of the primary antibody was visualized using the Novolink detection kit (Leica) and counterstained with diluted Mayers Haematoxylin (Surgipath). All sections were dehydrated (IDA), cleared in xylene, mounted with DPX (Leica biosystems), and observed using a Zeiss Axioskop 40. Images were captured with an Axiocam IcC5 using Zeiss Axiovision (version 4.8.2) as previously described.^19^

### 2.8 Western blot

Western blot analysis was performed using as previously described.^18^ Briefly, tissue samples were used from the different mesenteric fibrosis groups as described in Supporting Information S1. Protein was extracted from tissue samples (5 mg/sample) using 300 μL protein lysis RIPA buffer (Thermo Fisher) supplemented with 1:100 Protein Inhibitor cocktail (Sigma Aldrich), 0.5% NaF (Sigma Aldrich), 0.5% NaVan (Sigma Aldrich), and 1 mM PMSF (Sigma Aldrich). Protein concentrations were measured using the microBCA assay (Thermo Fisher). A total of 20 μg of protein was loaded into each well, and the samples were separated on 4%–12% SDS‐PAGE gels (Genscript) and transferred to PVDF membranes (Millipore). Membranes were incubated with either 5% Bovine Serum Albumin (BSA) or 5% milk for 1 h and incubated with primary antibodies overnight at 4°C (Supporting Information Table 3), followed by incubation with secondary antibodies for 1 h at room temperature. Protein expression was detected using SuperSignal West Pico Chemiluminescent Substrate (Thermo Fisher), and membranes were developed in a FluorChem M imager (ProteinSimple). When required, membranes were stripped for 15 min at 37°C with Restore Plus Stripping Buffer (Thermo Fisher Scientific). Band intensity was detected using ImageLab software 6.1 (Bio‐Rad Laboratories) following background subtraction and normalised to total protein staining using No‐stain labelling reagent (Invitrogen) as previously described.^20^

### 2.9 Statistics

Student's *t*‐test, which was applied in GraphPad Prism version 8, was used to compare proliferation and serotonin secretion between groups. RT^2^ Profiler PCR array data was analysed using the online platform provided by Qiagen (https://dataanalysis2.qiagen.com/pcr). All data have passed the quality control on the Qiagen platform. GraphPad Prism version 9 was used for statistical tests in qPCR and Western Blot analyses. A *p* value <.05 was used to demonstrate statistical significance.

## 3 RESULTS

### 3.1 Telotristat ethyl affects gene expression of stromal and SI‐NET cells

**FIGURE 1:**
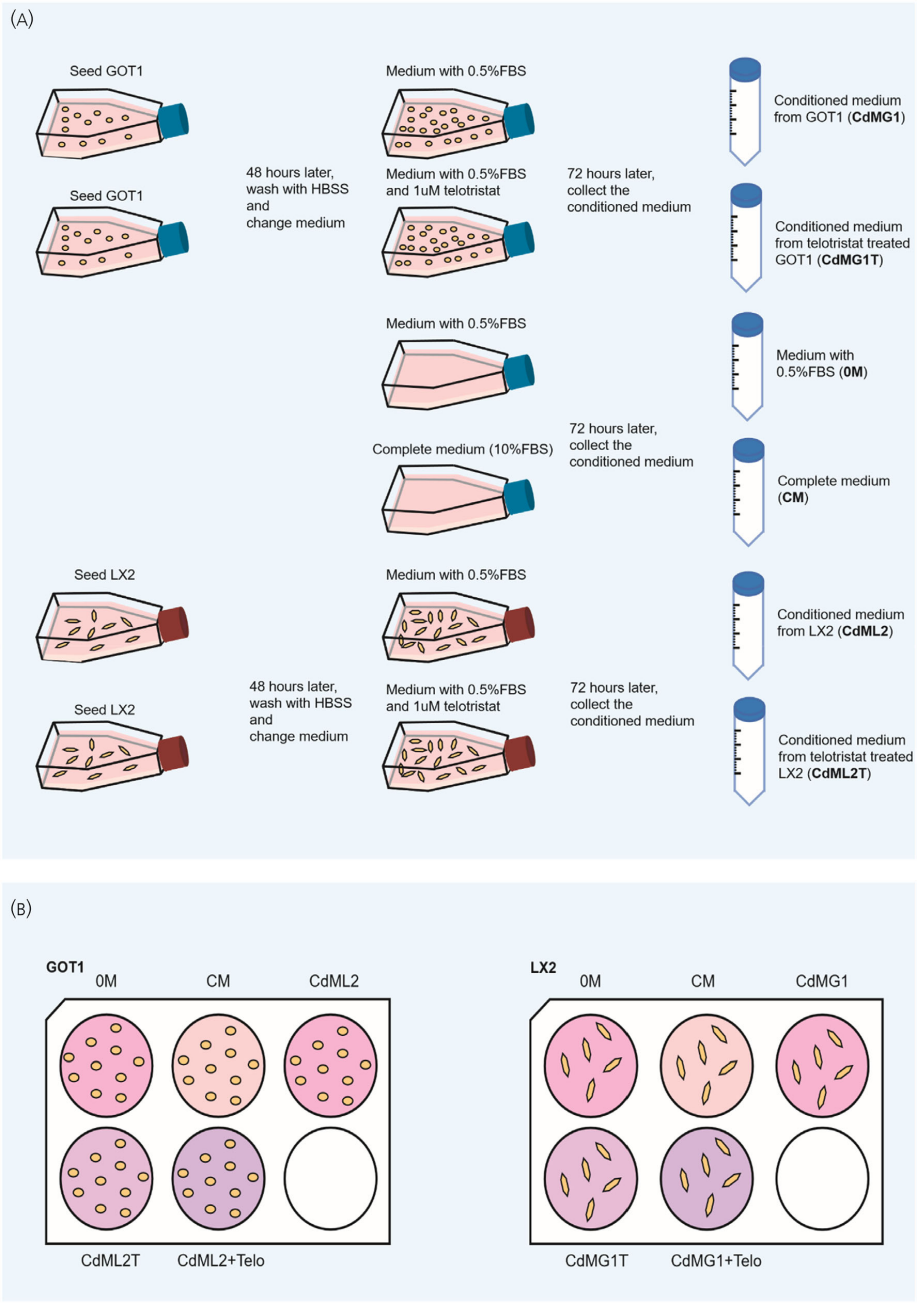
Protocol for conditioned medium paracrine in vitro model of GOT1 and LX2 cells. Protocol of making conditioned medium (A) and using conditioned medium to treat GOT1 and LX2 cells for RNA analysis (B). Conditioned media were collected according to Figure 2A and used to treat GOT1 and LX2 cells respectively for RNA analysis. CdMG1, conditioned medium from GOT1 cells; CdMG1T, conditioned medium from telotristat ethyl treated GOT1 cells; 0 M, medium with 0.5% FBS; CM, complete medium with 10% FBS; CdML2, conditioned medium from LX2 cells; CdML2T, conditioned medium from telotristat ethyl treated LX2 cells; CdML2 + Telo, conditioned medium from LX2 cells supplemented with telotristat ethyl; CdMG1 + Telo, conditioned medium from GOT1 cells supplemented with telotristat ethyl.

**FIGURE 2:**
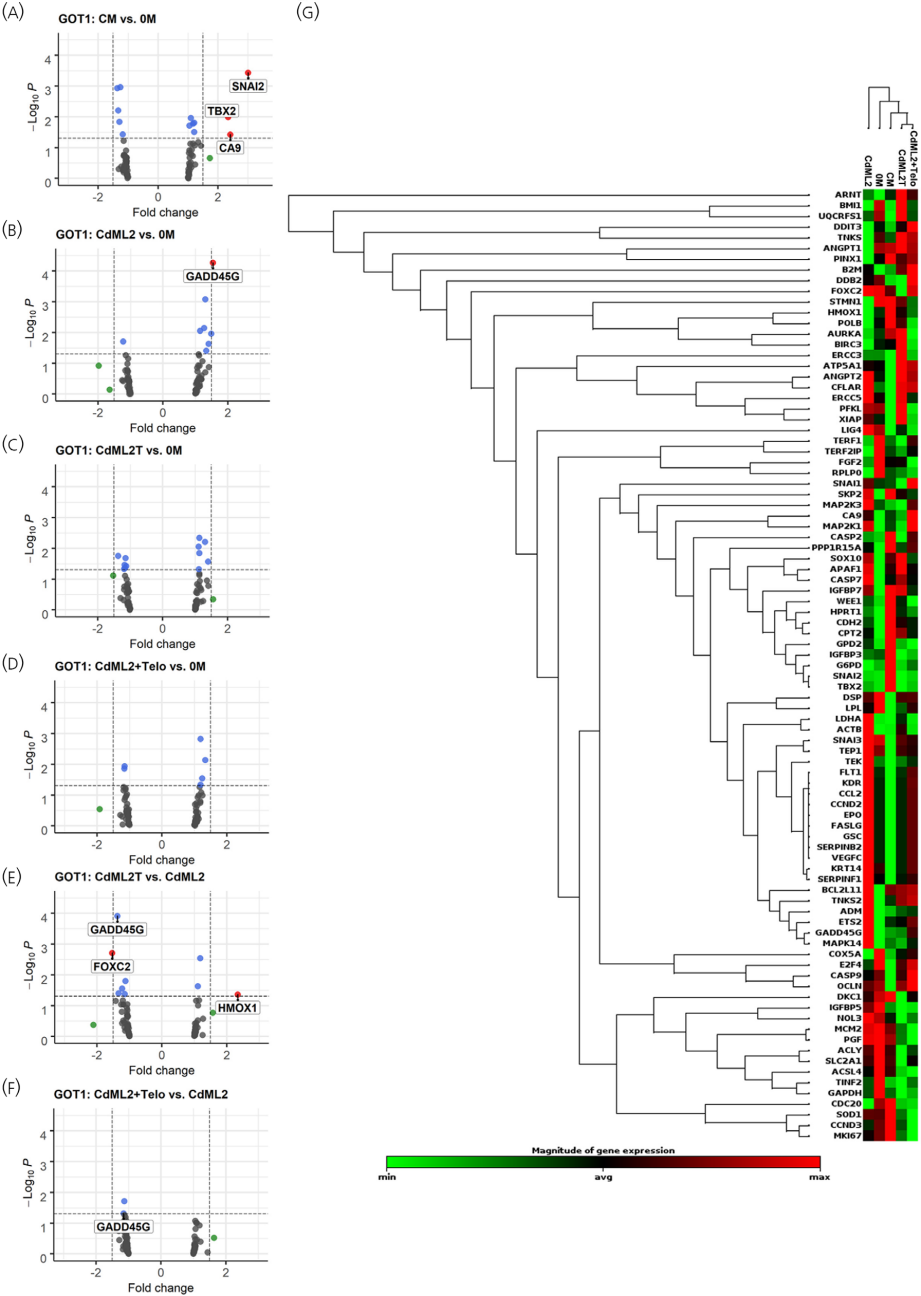
RT2 Profiler results of GOT1 cells using Human Cancer Pathway Finder Array. RT2 Profiler results of GOT1 cells (A–F) using Human Cancer Pathway Finder Array. Fold change (FC) of 1.5 and *p*‐value <.05 were set to find genes having significant changes between groups. Significantly changed genes were shown as red dots in the figure. CM, complete medium; 0 M, medium with 0.5% FBS; CdML2, conditioned medium from LX2 cells; CdML2T, conditioned medium from Telotristat ethyl treated LX2 cells; CdML2 + Telo, conditioned medium from LX2 cells supplemented with Telotristat ethyl; G. Clustergram of different groups.

To test the potential effect of TE on the crosstalk between SI‐NETs and fibroblasts, a co‐culture paracrine in vitro model was used (Figure 1). The RT2 Profiler of Human Cancer Pathway Finder array was performed using RNA extracted from GOT1 cells in the paracrine model. The data highlighted several genes including *GADD45G*, *FOXC2*, and *HMOX1* to be significantly affected in GOT1 cells that were treated with LX2 conditioned medium containing TE (CdML2T and CdML2+T) compared with GOT1 treated with LX2 medium without the presence of telotristat ethyl (0 M and CdML2), as shown in Figure 2 and Supporting Information Table 4. *GADD45G* is thought to be involved in stress signalling, cell cycle, and apoptosis control, which is commonly P53‐dependent,^21^ with previous studies suggesting the upregulation of GADD45G could be tumour‐suppressive.^22^, ^23^ Data from previous studies suggest that *HMOX1* might play different roles in many cancer pathways, including that the longer promoter GTn repeat polymorphism of *HMOX1* is associated with worse prognosis in pancreatic NET patients.^24^ *FOXC2*, a transcription factor, was found to be related to cell proliferation and epithelial‐mesenchymal transition (EMT) in cancer cells.^25^ As *FOXC2* was only downregulated in GOT1 cells treated with CdML2T, TE may be affecting the secreted proteins of LX2 cells, which subsequently impairs the expression of this gene in the tumour cells.

**FIGURE 3:**
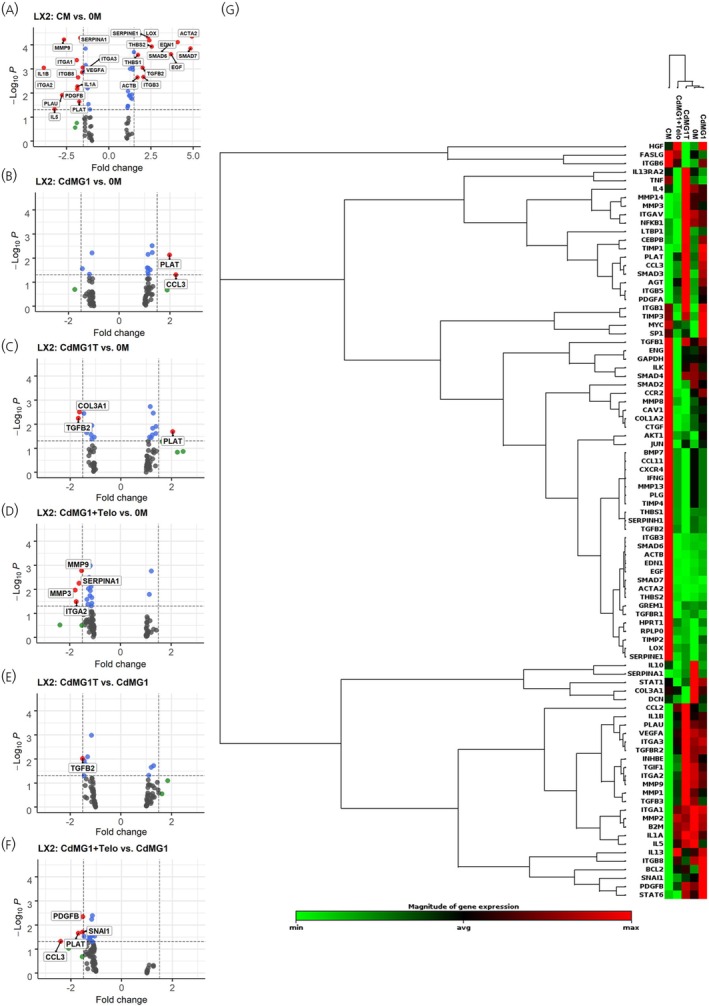
RT2 Profiler results of LX2 cells using Human Fibrosis Array. RT2 Profiler results of LX2 cells (G‐L) using Human Fibrosis Array. Fold change (FC) of 1.5 and *p*‐value <.05 were set to find genes having significant changes between groups. Significant changed genes were shown as red dots in the figure. CM, complete medium; 0 M, medium with 0.5% FBS; CdMG1, conditioned medium from GOT1; CdMG1T, conditioned medium from Telotristat ethyl treated GOT1; CdMG1 + Telo, conditioned medium from GOT1 cells supplemented with Telotristat ethyl; G. Clustergram of different groups.

For LX2 cells, known as stromal cells, the RT2 profiler of Human Fibrosis array was applied, which indicated two genes, *CCL3* and *PLAT*, were significantly upregulated when treated with conditioned medium of GOT1 cells (CdMG1) compared to 0.5% medium (0 M) but were downregulated when treated with conditioned medium from GOT1 cells supplemented with TE (CdMG1 + Telo; Figure 3F). This could be indicative of a direct effect of TE on the expression of these genes within the stromal cells and could negate the crosstalk from the tumour cells, which appears to increase their expression. *PLAT*, a secreted serine protease, has been demonstrated to enhance cancer cell migration and invasion,^26^ whilst *CCL3*, a gene coding for a ligand for CC motif chemokine receptor 1 and 5 (CCR1 and CCR5), has been implicated in promoting tumour growth.^27^ Overall, this small set of data showed the potential effect of TE on the crosstalk between SI‐NET cells and stromal cells by significantly changing several cancer‐related and fibrosis gene targets, respectively.

### 3.2 Identification of enriched signalling pathways and generation of first tier gene targets

**FIGURE 4:**
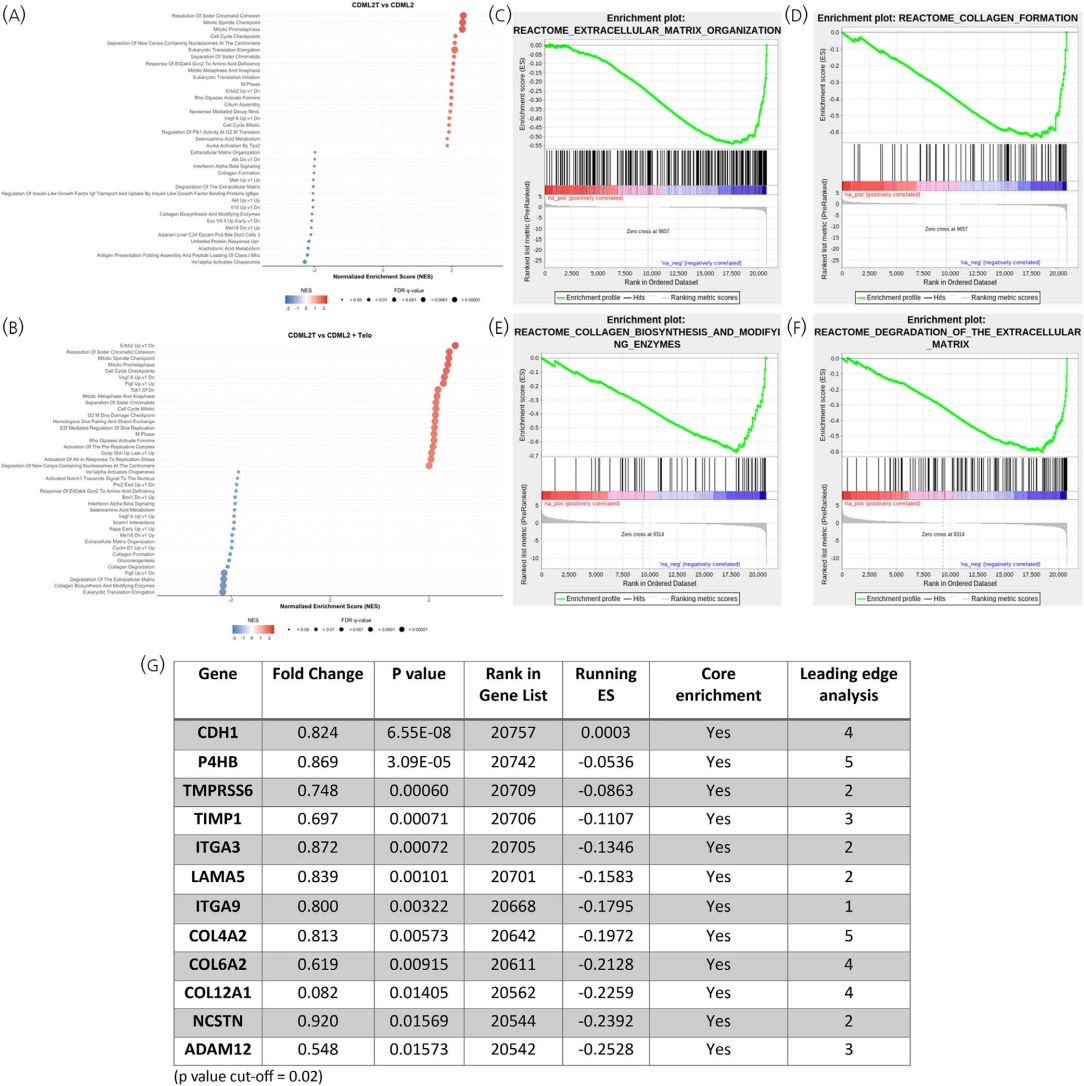
GSEA results from the comparisons between CDML2T vs. CDML2 and CDML2T vs. CDML2 + Telo. Gene set enrichment analysis (GSEA) of RNA sequencing data. (A) Top 20 positively and negatively enriched reactomes in the comparison between CDML2T and CDML2. (B) Top 20 positively and negatively enriched reactomes in the comparison between CDML2T and CDML2 + Telo. (C, D) Examples of enrichment plots of ECM‐related reactomes in the comparison between CDML2T and CDML2. (E, F) Examples of enrichment plots of ECM‐related reactomes in the comparison between CDML2T and CDML2 + Telo. (G) Table with a summary of core‐enriched genes within the ECM‐related reactomes from the comparison between CDML2T and CDML2.

RNA‐sequencing was performed followed by GSEA to determine the enrichment of reactomes in the different conditions. Conditioned medium had minimal effects on GOT1 (CdML2toG1 vs. 0 M) and LX2 (CdMG1toL2 vs. 0 M) cells, showing only one and five enriched reactomes, respectively, mainly indicating suppressed lipid metabolism in LX2 cells (Supporting Information Figure S2A,C). In LX2 cells, indirect telotristat ethyl treatment (CdMG1TtoL2) enriched KRAS signalling and interleukin pathways while downregulating cholesterol biosynthesis compared to conditioned medium (Supporting Information Figure S2D), whereas direct treatment (CdMG1toL2 + Telo) suppressed interferon and KRAS pathways (Supporting Information Figure S2E). Comparing indirect and direct treatments in LX2 cells showed similar activation of KRAS and immune pathways as seen with the indirect versus conditioned medium alone comparison along with the suppression of lipid metabolism (Supporting Information Figure S2F). In GOT1 cells, indirect treatment (CdML2TtoG1 vs. CdML2toG1) enriched cell cycle pathways including the mitotic spindle checkpoint (Figure 4A), while direct treatment (CdML2toG1 + Telo vs. CdML2toG1) enriched translation and amino acid metabolism but suppressed DNA replication and cell cycle checkpoints (Supporting Information Figure S2B). Comparing indirect versus direct treatment showed similar enrichment of cell cycle and mitotic pathways, with negative enrichment of translation (Figure 4B). When focusing on reactomes involved in the extracellular matrix (ECM) that could be involved in the development of fibrosis, the GSEA data showed negative enrichment of ECM‐related reactomes in GOT1 cells cultured with the conditioned medium of LX2 cells treated with TE (CdML2TtoG1) compared with GOT1 cells treated with the conditioned medium of LX2 cells only (CdML2toG1) (Figure 4A). Moreover, the same downregulation of these pathways was observed when comparing GOT1 cells cultured with the conditioned medium of LX2 cells treated with TE (CdML2TtoG1) against GOT1 cells treated with the conditioned medium of LX2 cells which were then supplemented with Telotristat (CdML2toG1T), indicating an indirect action of TE on the ECM‐related reactomes (Figure 4B). These reactomes included extracellular matrix organisation (False Discovery Rate (FDR)—0.049714245), degradation of the extracellular matrix (FDR—0.04200256), collagen biosynthesis and modifying enzymes (FDR—0.041578107), and collagen formation (FDR—0.047106877) (Figure 4C–F). A list of target genes was generated based on core genes from these enriched reactomes, leading edge analysis, RNA‐sequencing data, and current literature which is shown in Figure 4G. *CDH1* is thought to act as a tumour‐suppressor gene with the loss of gene function contributing to cancer progression by increasing proliferation, invasion, and metastasis.^28^, ^29^ *P4HB* encodes an enzyme involved in hydroxylation of prolyl residues in preprocollagen and studies have highlighted this gene as a potential novel biomarker for bladder carcinoma^30^ or prognostic marker for gastric cancer.^31^ *TMPRSS6* encodes for a protein involved in ECM remodelling/degradation processes of including type I collagen, fibronectin, and fibrinogen^32^ and may be an important factor in the development of breast cancer.^33^ *TIMP1* encodes for a peptidase involved in the degradation of the extracellular matrix and has been shown to be upregulated in breast and gastric cancer,^34^ whilst also being highlighted as a potential biomarker for colorectal cancer.^35^ *ITGA3* encodes for a protein that forms part of an integrin complex and is a potential diagnostic and prognostic marker in pancreatic cancer^36^ and a potential therapeutic target in ovarian cancer.^37^ *LAMA5* encodes for an extracellular matrix glycoprotein and has been suggested as a promoter of liver metastases during colorectal cancer.^38^ *COL4A2* encodes for a protein that is a structural component of basement membranes and a potential biomarker for liver^39^ and gastric cancers.^40^ *COL6A2* encodes for a protein that is a beaded filament protein found in connective tissues and upregulation of *COL6A2* has been linked with a worse prognosis for bladder cancer.^41^ *COL12A1* encodes for a protein that is a fibril‐associated collagen and is a potential prognostic and therapeutic target in gastric cancer.^42^ *NCSTN* is the gene for a type I transmembrane glycoprotein that has been demonstrated to promote the progression of liver cancer^43^ and be a potential biomarker for ovarian cancer. *ADAM12* encodes for a protein that has been implicated in a variety of biological processes involving cell–cell and cell‐matrix interactions, is upregulated in epithelial cancers and is a potential biomarker for pancreatic cancer.^44^

### 3.3 Expression profiles of target genes in human SI‐NET tissues

The GSEA analysis of in vitro data so far indicated that the expression of the target genes is downregulated when grown in conditioned medium with TE. Therefore, to further confirm their involvement in tumour progression and mesenteric fibrosis, several target genes were analysed in human tissue samples from SI‐NET patients.

**FIGURE 5:**
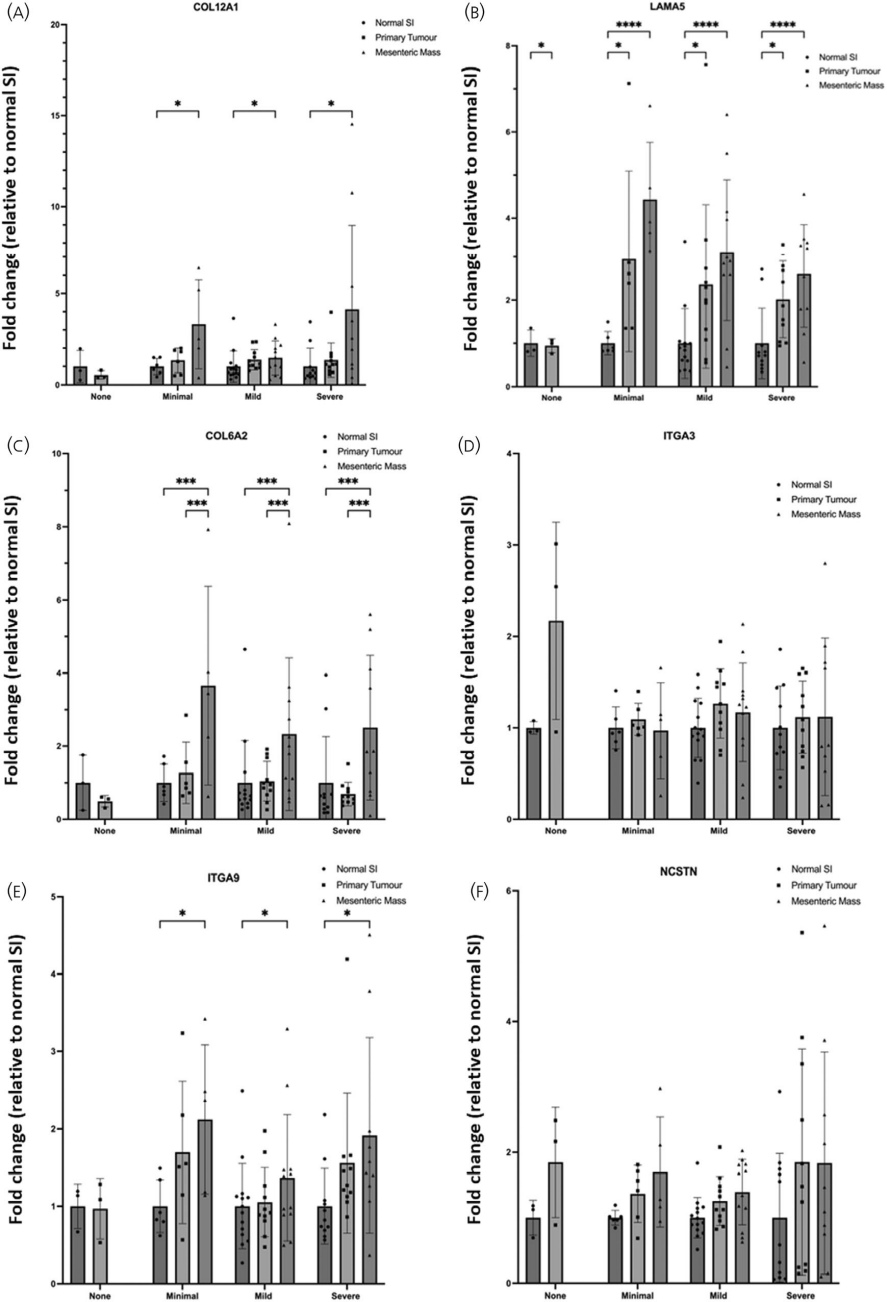
Gene expression profile of core enriched genes in human SI‐NET tissue. Gene expression profile of core enriched genes (A) COL12A1, (B) LAMA5, (C) COL6A2, (D) ITGA3, (E) ITGA9, and (F) NCSTN in human SI‐NET tissue. Patients were grouped into four categories of fibrosis: None (*n* = 3), Minimal (normal SI *n* = 6, primary tumour *n* = 6, mesenteric mass *n* = 5), Mild (normal SI *n* = 14, primary tumour *n* = 11, mesenteric mass *n* = 12), and Severe (normal SI *n* = 11, primary tumour *n* = 11, mesenteric mass *n* = 10). A two‐way ANOVA was performed, and adjusted *p* values are shown after correction for multiple comparisons (*p(adj) <.05, **p(adj) <.01, ***p(adj) <.001, ****p(adj) <.0001).

*COL12A1* (Figure 5A) showed significantly increased expression in the mesenteric metastases compared to the normal SI in each of the fibrotic groups. Both the primary tumour and the mesenteric metastases showed significant upregulation of *LAMA5* (Figure 5B) within the different groups of patients with fibrosis, whilst the patients with no fibrosis showed a significant downregulation in the primary tumour compared to the normal SI. There were no significant differences between the tissue types and the different categories of fibrosis for *ITGA3* (Figure 5C). *COL6A2* (Figure 5D) demonstrated significant upregulation across the different categories of fibrotic patients in the mesenteric metastases compared to both the primary tumour and normal SI. Like *COL12A1*, the expression of *ITGA9* was significantly increased in the mesenteric metastases compared to the normal SI (Figure 5E). Whilst there were no significant differences of expression between the groups for *NCSTN* (Figure 5F), there was a consistent trend of increased expression in the primary tumour and mesenteric metastases compared to the normal SI in each of the fibrotic groups.

### 3.4 Proteins expressed by target genes in human SI‐NET tissues were localised in tumour or stroma

**FIGURE 6:**
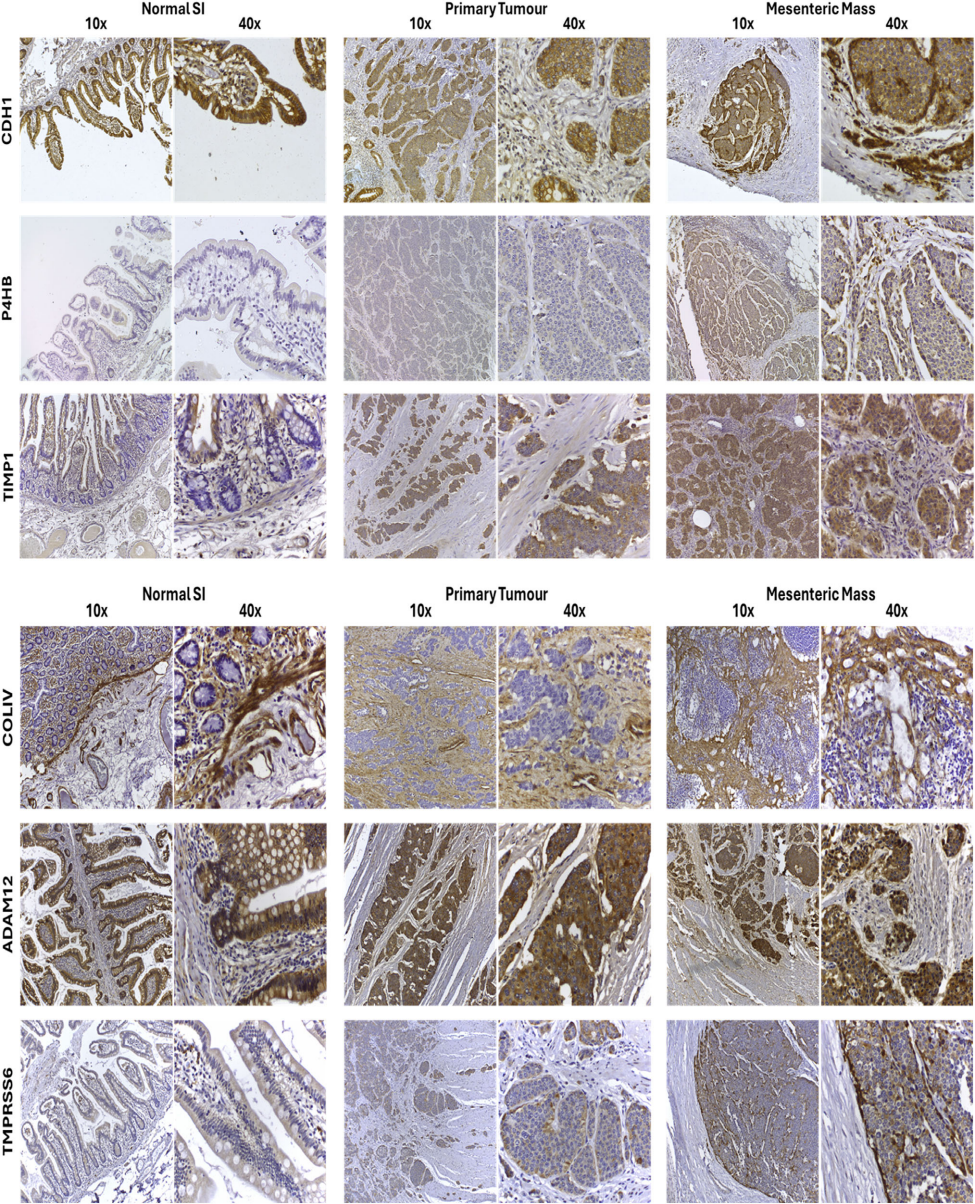
Distribution and expression of proteins involved in tumour–stromal crosstalk in patients with SI‐NETs. Distribution and expression of proteins involved in microenvironment, tumour–stromal crosstalk in patients with SI‐NETs. (A) Immunohistochemistry was performed on matched samples of normal tissue, primary tumour tissue, and mesenteric mass. CDH1 expression of a patient with an advanced fibrotic midgut NET. P4HB expression of a patient with advanced mesenteric fibrosis. TIMP1 expression of a patient with mild fibrosis. COL4 expression of a patient with mild fibrosis. ADAM12 expression of a patient with advanced mesenteric fibrosis. TMPRSS6 expression of a patient with advanced mesenteric fibrosis (all images were taken at 10× and 40× magnification).

The localization of several of the target genes was further investigated by immunohistochemistry in the tissue of 10 patients (3 patients with no fibrosis, 2 patients with minimal fibrosis, 2 patients with mild fibrosis, and 3 patients with severe fibrosis). This was assessed in paired normal SI, primary tumour, and mesenteric metastases, and representative examples are displayed in Figure 6. CDH1 showed strong, diffuse membranous expression and weak cytoplasmic staining in primary tumour cells. In the samples of mesenteric metastases with severe fibrosis, CDH1 stained more strongly at the periphery of the tumour nests, which were in contact with the stroma. Tumour cells in the centre of the tumour nests showed cytoplasmic positivity, often including a reduction in staining at the plasma membrane. P4HB was not expressed in any of the primary tumours of patients that had severe mesenteric fibrosis, but it was expressed in both the tumour cell cytosol and the surrounding stromal cells in some of the mesenteric metastases. P4HB showed none, weak, or moderate staining in the primary tumours and mesenteric masses of patients with minimal or mild fibrosis, often with the most intense staining at the periphery of the tumour nests. All tumour cells expressed TIMP1 in the cytosol to varying levels in the primary tumours and the paired metastatic mesenteric masses. Collagen IV was expressed within the stromal ECM between all primary tumours and metastatic metastases. The intensity varied, with either the primary tumour or the mesenteric metastases showing less intense staining than the other. ADAM12 was expressed by all primary tumours and was detectable in the cytosol of tumour cells in the mesenteric metastases. ADAM12 was also present in the stromal ECM surrounding some of the ADAM12 positive tumours. Stromal ECM and stromal cells were negative for TMPRSS6 but were weakly expressed in the cytosol of primary tumour cells, with intense staining of small clusters of cells that were in contact with the surrounding stroma.

### 3.5 Gene and protein expression profiles of second tier genes in human SI‐NET tissues

Next, a panel of second tier targets was created, based on the results from the RT2 Profiler, a literature search and STRING analysis,^45^ focusing on downstream effectors and signalling pathways of the original target genes.

**FIGURE 7:**
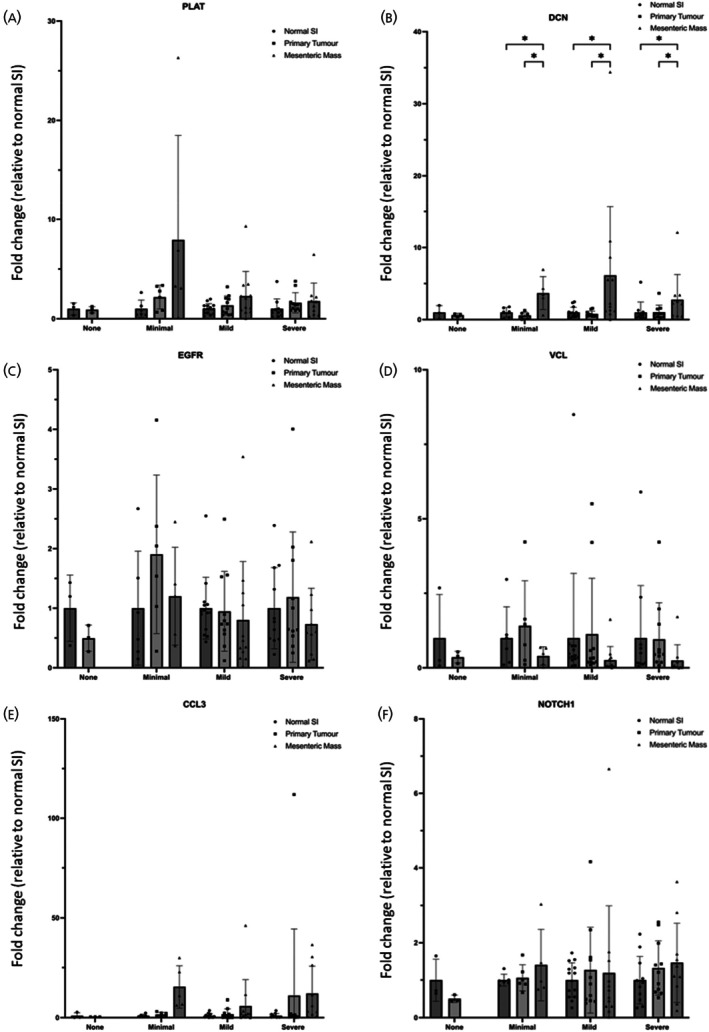
Gene expression profile of second tier genes from RT2 Profiler, STRING analysis and literature. Gene expression profile of second tier genes (A) PLAT, (B) DCN, (C) EGF‐R, (D) VCL, (E) CCL3, and (F) NOTCH1 from RT2 Profiler, STRING analysis, and literature. Patients were grouped into four categories of fibrosis: None (*n* = 3), Minimal (normal SI *n* = 6, primary tumour *n* = 6, mesenteric mass *n* = 5), Mild (normal SI *n* = 14, primary tumour *n* = 11, mesenteric mass *n* = 12), and Severe (normal SI *n* = 11, primary tumour *n* = 11, mesenteric mass *n* = 10). A two‐way ANOVA was performed, and adjusted *p* values are shown after correction for multiple comparisons (*p(adj) <.05).

RT‐qPCR was performed on several of these targets using the same cohort of patients as described above. Again, there were no significant changes between the normal SI and primary tumour of patients with no fibrosis (Figure 7). *PLAT* expression (Figure 7A) had a tendency of increased expression in the primary tumour and mesenteric when compared to the normal SI in each category of fibrosis but lacked significance. The expression of *DCN* (Figure 7B) was significantly upregulated in the mesenteric mass compared to both the normal SI and primary tumour tissue of patients in each fibrotic category. *EGFR* and *VCL* expression (Figure 7C,D) showed a tendency to exhibit an increased expression in the normal SI and primary tumour compared to the mesenteric mass in each category of fibrosis; however, these did not reach significance. The expression pattern between the different groups seen in the expression of *CCL3* (Figure 7E) was similar to that of *DCN*, with the addition of tumour tissue of the severely fibrotic patients being upregulated compared to the normal SI, albeit lacking significance. A similar trend was observed for *NOTCH1* expression (Figure 7F), which showed increased expression in minimal fibrosis in comparison to mild and severe fibrosis, but without reaching significance.

**FIGURE 8:**
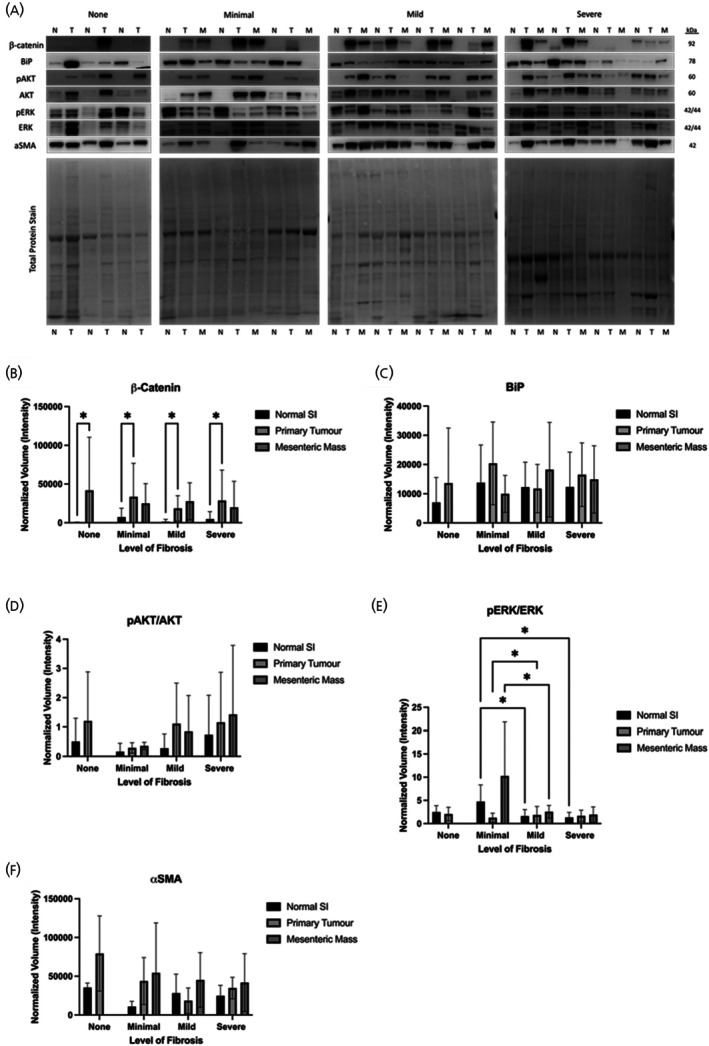
Protein expression on human SI‐NET tissue. Protein expression on human SI‐NET tissue. Western blot analysis was performed on protein from patients grouped into four categories of fibrosis: None (*n* = 3), Minimal (normal SI *n* = 6, primary tumour *n* = 6, mesenteric mass *n* = 5), Mild (normal SI *n* = 14, primary tumour *n* = 11, mesenteric mass *n* = 12) and Severe (normal SI *n* = 11, primary tumour *n* = 11, mesenteric mass *n* = 10). (A) Representative Western blots for each fibrosis group are shown. Densitometry analysis was performed for (B) β‐catenin, (C) BiP, (D) pAKT/AKT ratio, (E) pERK/ERK ratio and (F) αSMA. Total protein loading was used for normalization. A two‐way ANOVA was performed, and adjusted *p* values are shown after correction for multiple comparisons (*p(adj) <.05). αSMA, α‐smooth muscle actin; SI, small intestine.

Western Blot analysis was performed on another set of the second tier targets, β‐catenin, Binding immunoglobulin protein (BiP), pAKT/AKT, pERK/ERK, and αSMA, to validate their presence and expression in the human tissue. These results were semi‐quantified to determine the levels of expression between the normal SI, primary tumour, and mesenteric mass of the patients. Figure 8A shows a representation of the Western blot membranes that were performed for each fibrotic group, including the total protein stain that was used for normalization. β‐catenin (Figure 8B) was significantly upregulated in the primary tumour tissue compared to the normal SI for patients of each category, including the patients with no fibrosis. BiP (Figure 8C) showed no significant differences or tendencies between the different conditions investigated. The ratio of pAKT/AKT (Figure 8D) tended to increase between the different tissue types of each category of fibrosis, and the intensity was higher as the severity of the fibrosis increased; however, this lacked statistical significance. Protein expression of pERK/ERK (Figure 8E) showed a significant decrease in normal tissue of mild and severe fibrosis when compared with minimal fibrosis, whereas the primary tumour of mild fibrosis showed a significant increase versus the primary tumour of minimal, with the mesenteric mass of minimal being significantly upregulated versus mild mesenteric mass. Apart from the primary tumour tissue in mildly fibrotic patients, αSMA (Figure 8F) showed a tendency to increase in primary tumour tissue and mesenteric mass of patients for each category of fibrosis.

## 4 DISCUSSION

Despite several studies highlighting the possible anti‐tumour activity of TE in SI‐NETs,^9^, ^10^ the effect is yet to be robustly investigated in in vitro models specifically representing SI‐NETs. There is a distinct lack of available models and cell lines in the study of SI‐NETs, with many of the cell lines previously used for research no longer expressing the key markers of an SI‐NET cell or being wrongly identified as SI‐NET. Hofving et al. showed by performing immunophenotyping, copy number profiling, whole‐exome sequencing, and a large‐scale inhibitor screening of seven GEPNET cell lines that the GOT1 cells, derived from an SI‐NET liver metastasis, maintained an SI‐NET phenotype, including a loss of chromosome 18, wild type TP53, and expression of SSTR2, and thus are one of the best SI‐NET cell lines available.^15^ Given that a literature search on TE and specifically SI‐NET and GOT1 cells yielded no results, this study represents the first analysis of TE on SI‐NET cells in vitro.

The paracrine in vitro model used for this study was previously used by our group to identify the interactions between SI‐NET cells and stromal cells^18^ and as such was used again in this study to highlight both the direct and indirect action of the telotristat ethyl. Mesenteric fibrosis, defined by a fibrotic reaction to a metastatic lesion in the mesentery, affects up to 50% of SI‐NET patients and causes severe complications and a shorter overall survival, as previously shown by our team.^46^, ^47^ Based on the data obtained from the RNA‐sequencing and subsequent GSEA, extracellular matrix‐related reactomes in GOT1 cells are indirectly affected by the telotristat ethyl. Furthermore, several of the highlighted genes from the enriched reactomes were upregulated in the tumour and mesenteric metastases in our cohort of 34 SI‐NET patients stratified in subgroups with varying levels of mesenteric fibrosis. Analysis of downstream effectors of the first‐tier targets in our second‐tier group showed significant increases in β‐catenin in the primary tumour tissue. The NCSTN‐Notch1‐AKT pathway has been shown to regulate the activation and nuclear translocation of Beta Catenin (CTNNB1) which promotes the invasive capacity of HCC cells.^43^ Several studies have also demonstrated an accumulation of β‐catenin in NET cell lines and that inhibiting β‐catenin resulted in a reduction in cell growth.^48^, ^49^ Our study has demonstrated a strong significant effect on downregulation of ITGA3 in severe fibrosis versus mild fibrosis, whereas ITGA9 showed a significant effect in all mesenteric mass versus normal SI in all fibrotic groups. Integrin‐mediated interactions are vital to the maintenance of normal cell functioning because of their ability to mediate signalling and a previous study from our group has suggested that the integrin signalling pathway may be important in the pathogenesis of SI‐NETs.^18^

In this study, several of the ECM‐related genes downregulated by telotristat in the paracrine in vitro model were shown to be upregulated in the mesenteric metastasis in the tissue of SI‐NET patients. Given that the pathogenesis of mesenteric fibrosis is poorly understood, these results suggest that telotristat ethyl could also have an impact on the development of this fibrosis and further research could lead to better understanding with the possibility of telotristat ethyl being used as an anti‐fibrotic agent. A recent study reported reduced expression of serotonin‐metabolizing enzymes in the mesenteric metastases of patients with fibrosis.^50^ Taken together with our findings, this suggests that the potential anti‐fibrotic effect of telotristat may be due to its ability to prevent serotonin accumulation within the mesenteric metastases of SI‐NET patients.

Previous studies have shown that an anti‐tumour effect of TE is seen in in vivo mouse models^51^, ^52^; however, other studies have suggested that the potential anti‐proliferative effect of TE does not occur in both 2D and 3D in vitro monocultures of pancreatic NET cell lines.^12^ Therefore, it is reasonable to suggest that a disruption in the interactions between tumour and stromal cells in the presence of TE is a potential mechanism for the anti‐tumour activity seen in SI‐NETs. To our knowledge, this study also represents the first analysis of TE on the crosstalk between SI‐NET cells (GOT1 cells) and stromal cells. Interestingly, we observed unexpected positive enrichment of cell cycle–related pathways in tumour cells following indirect telotristat treatment and conditioned medium from stromal cells, whereas direct treatment showed the opposite effect. As this study focused primarily on fibrotic pathways, further work exploring other components of the tumour microenvironment will be needed to clarify the mechanisms behind this unexpected cell cycle activation and the broader anti‐cancer effects.

Despite our study highlighting several genes involved in the interactions between tumour and stromal cells in SI‐NETs which are altered in the presence of telotristat ethyl, there are several limitations. Firstly, although GOT1 cells express neuroendocrine markers and are generally considered one of the most representative SI‐NET cell lines,^15^ this cell line is derived from a liver metastasis of an SI‐NET patient, whilst the LX2 cell line is derived from liver stromal cells. Thus, this model may not be a true representation of the interactions between NET cells and fibroblasts of intestinal origin but more of SI‐NET‐induced liver metastasis. Secondly, whilst being able to suggest pathways or genes which are altered in the presence of telotristat ethyl, the paracrine in vitro model does not account for the much more complex tumour microenvironment containing many cell types and the physical or direct interactions between them. Nevertheless, the well‐classified patient cohort enabled us to confirm that the highlighted genes from the in vitro model were differentially expressed across different tissue types and levels of fibrosis. However, we lack data of a patient cohort who had been treated with telotristat pre‐surgery which would have enabled us to validate the effect of telotristat in the tumour or metastatic tissue. Nevertheless, clinical studies such as TELEACE, a longitudinal analysis, showed an 8.5% reduction in tumour size associated with TE.^9^, ^10^

In conclusion, this study represents the first steps to determining the effect of telotristat ethyl on the interactions between SI‐NET cells and stromal cells. More research and further validation are needed to better understand the potential anti‐tumour and anti‐fibrotic activity of telotristat, which could lead to better treatment options for SI‐NET patients.

## AUTHOR CONTRIBUTIONS

**Harry Hodgetts:** Methodology; data curation; investigation; validation; formal analysis; visualization; writing – original draft; writing – review and editing. **Maria Castanho Martins:** Methodology; data curation; investigation; validation; formal analysis; visualization; writing – review and editing. **Luohai Chen:** Methodology; data curation; investigation; formal analysis; visualization. **Andrew R. Hall:** Methodology; validation; formal analysis. **Tu Vinh Luong:** Methodology; validation; formal analysis. **Dalvinder Mandair:** Methodology. **Martyn Caplin:** Conceptualization; funding acquisition; supervision; writing – review and editing. **Krista Rombouts:** Conceptualization; funding acquisition; project administration; supervision; writing – review and editing.

## FUNDING INFORMATION

This research was funded by a grant from IPSEN INNOVATION S.A.S. and by the UCL NIHR Biomedical Research Centre BRC648.III.KR.101350 (Krista Rombouts).

## CONFLICT OF INTEREST STATEMENT

There is no conflict of interest that could be perceived as prejudicing the impartiality of the research reported. Krista Rombouts owns shares in Engitix Therapeutics Ltd. and receives consultancies from Engitix Therapeutics Ltd. Tu Vinh Luong receives consultancies from Engitix Therapeutics Ltd.

## PEER REVIEW

The peer review history for this article is available at https://www.webofscience.com/api/gateway/wos/peer‐review/10.1111/jne.70094.

## ETHICS STATEMENT

The study was approved by the UCL Biobank Ethical Review Committee (reference number NC2017.003) and all patients signed informed written consent.

## Supporting information


**Figure S1.** Optimization of culture conditions, FBS and Telotristat ethyl concentrations for in vitro experiments. A, B. Cell morphology of GOT1 (A) and LX2 cells (B). GOT1 cells are small round‐shaped cells which grow as “colonies” with a long doubling time of more than 5 days. LX2 cells are star‐shaped, larger than GOT1 cells in size and grow much faster than GOT1 cells. C, D. Proliferation effects of different concentrations of FBS to GOT1 cells (C) and LX2 cells (D) with *n* = 6 for each condition; ns indicates not statistically significant; *p* <.05 indicates statistically significant. E‐F. Proliferation effects of different concentrations of telotristat ethyl to GOT1 cells (E) and LX2 cells (F) with *n* = 6 for each condition; ns indicates not statistically significant; *p* <.05 indicates statistically significant. G‐H. Effects of telotristat ethyl to GO1 cells (G) and LX2 (H) in inhibiting serotonin secretion with *n* = 3 for each condition; *p* <.05 indicates statistically significant.
**Figure S2**. GSEA data of the RNA sequencing of the cells within the paracrine in vitro model. Gene set enrichment analysis of RNA sequencing. The top 20 positively and negatively enriched reactomes in comparisons between (A) CDML2 vs. 0 m, (B) CDML2 + Telo vs. CDML2, (C) CDMG1 vs. 0 M, (D) CDMG1T vs. CDMG1, (E) CDMG1 + Telo vs. CDMG1 and (F) CDMG1T vs. CDMG1 + Telo.

**Table S1.** Summary of demographic and clinical characteristics of patients enrolled in the study.
**Supporting Information Table 1**. Assay‐on‐Demand primers used for RT‐qPCR.
**Supporting Information Table 2**. Summary of primary antibodies, pre‐treatments and dilutions used for immunohistochemistry.
**Supporting Information Table 3**. Summary of primary antibodies, hosts and dilutions used for western blot analysis.
**Supporting Information Table 4**. Significantly changed genes between groups using RT2 profiler.

## Data Availability

The data that support the findings of this study are available from the corresponding author upon reasonable request.
