# Supplementary material for: Telotristat ethyl affects tumour‐fibroblast crosstalk in small intestinal neuroendocrine tumours
Source: J Neuroendocrinol. 2025 Sep 25;37(11):e70094. doi: 10.1111/jne.70094 (PMC12580468; doi:10.1111/jne.70094)
Supplement: Supplementary file 1 — Figure S1. Optimization of culture conditions, FBS and Telotristat ethyl concentrations for in vitro experiments. A, B. Cell morphology of GOT1 (A) and LX2 cells (B). GOT1 cells are small round‐shaped cells which grow as “colonies” with a long doubling time of more than 5 days. LX2 cells are star‐shaped, larger than GOT1 cells in size and grow much faster than GOT1 cells. C, D. Proliferation effects of different concentrations of FBS to GOT1 cells (C) and LX2 cells (D) with n = 6 for each condition; ns indicates not statistically significant; p <.05 indicates statistically significant. E‐F. Proliferation effects of different concentrations of telotristat ethyl to GOT1 cells (E) and LX2 cells (F) with n = 6 for each condition; ns indicates not statistically significant; p <.05 indicates statistically significant. G‐H. Effects of telotristat ethyl to GO1 cells (G) and LX2 (H) in inhibiting serotonin secretion with n = 3 for each condition; p <.05 indicates statistically significant. Figure S2. GSEA data of the RNA sequencing of the cells within the paracrine in vitro model. Gene set enrichment analysis of RNA sequencing. The top 20 positively and negatively enriched reactomes in comparisons between (A) CDML2 vs. 0 m, (B) CDML2 + Telo vs. CDML2, (C) CDMG1 vs. 0 M, (D) CDMG1T vs. CDMG1, (E) CDMG1 + Telo vs. CDMG1 and (F) CDMG1T vs. CDMG1 + Telo. [file JNE-37-e70094-s001.docx]

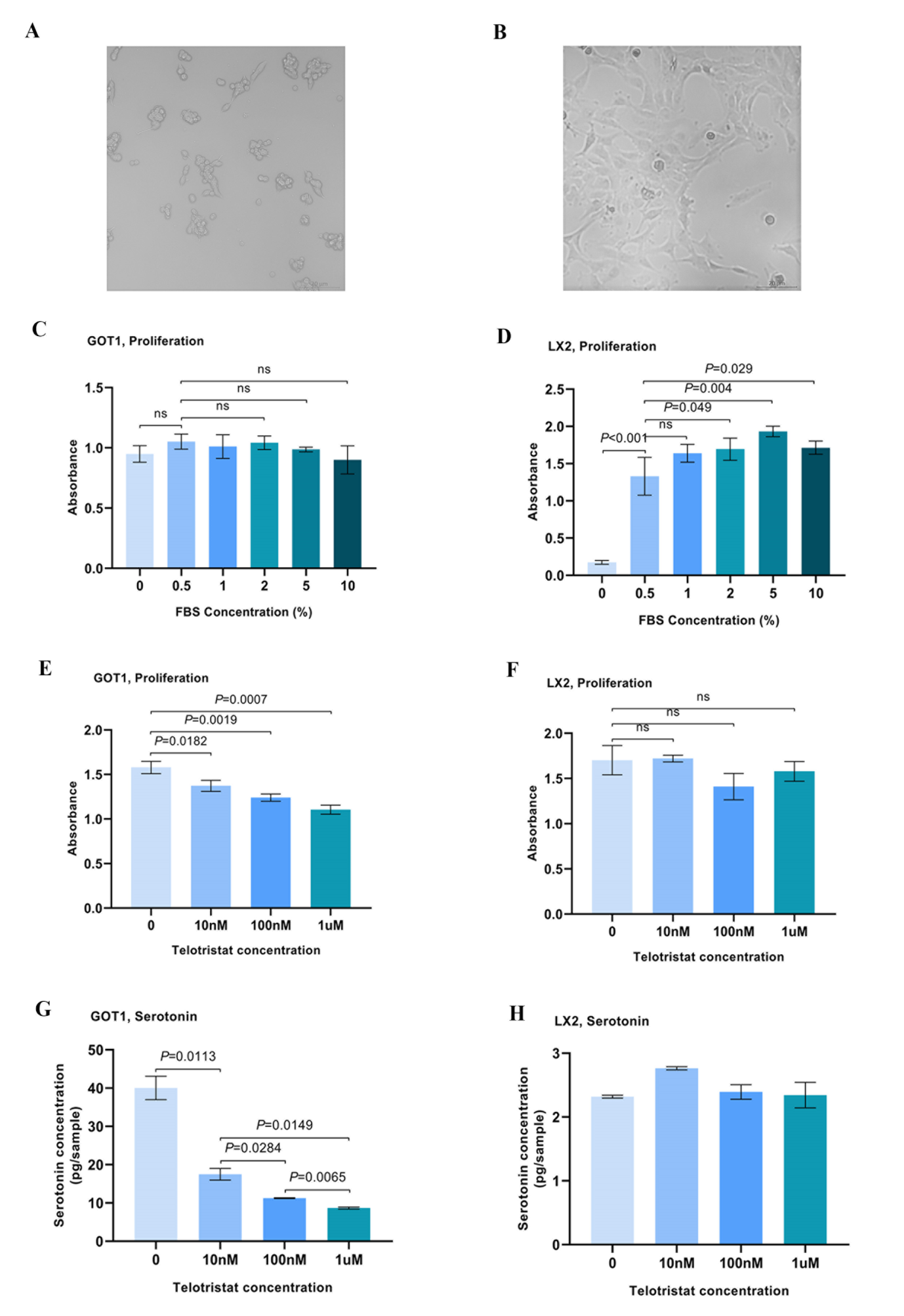
**Supplementary Figure 1. Optimization of culture conditions, FBS and Telotristat ethyl concentrations for *in vitro* experiments.**

**Supplementary Figure 1.** A-B. Cell morphology of GOT1 (A) and LX2 cells (B). GOT1 cells are small round-shaped cells which grow as “colonies” with a long doubling time of more than 5 days. LX2 cells are star-shaped, larger than GOT1 cells in size and grow much faster than GOT1 cells. C-D. Proliferation effects of different concentrations of FBS to GOT1 cells (C) and LX2 cells (D) with n=6 for each condition; ns indicates not statistically significant; *P*<0.05 indicates statistically significant. E-F. Proliferation effects of different concentrations of telotristat ethyl to GOT1 cells (E) and LX2 cells (F) with n=6 for each condition; ns indicates not statistically significant; *P*<0.05 indicates statistically significant. G-H. Effects of telotristat ethyl to GO1 cells (G) and LX2 (H) in inhibiting serotonin secretion with n=3 for each condition; *P*<0.05 indicates statistically significant.

**
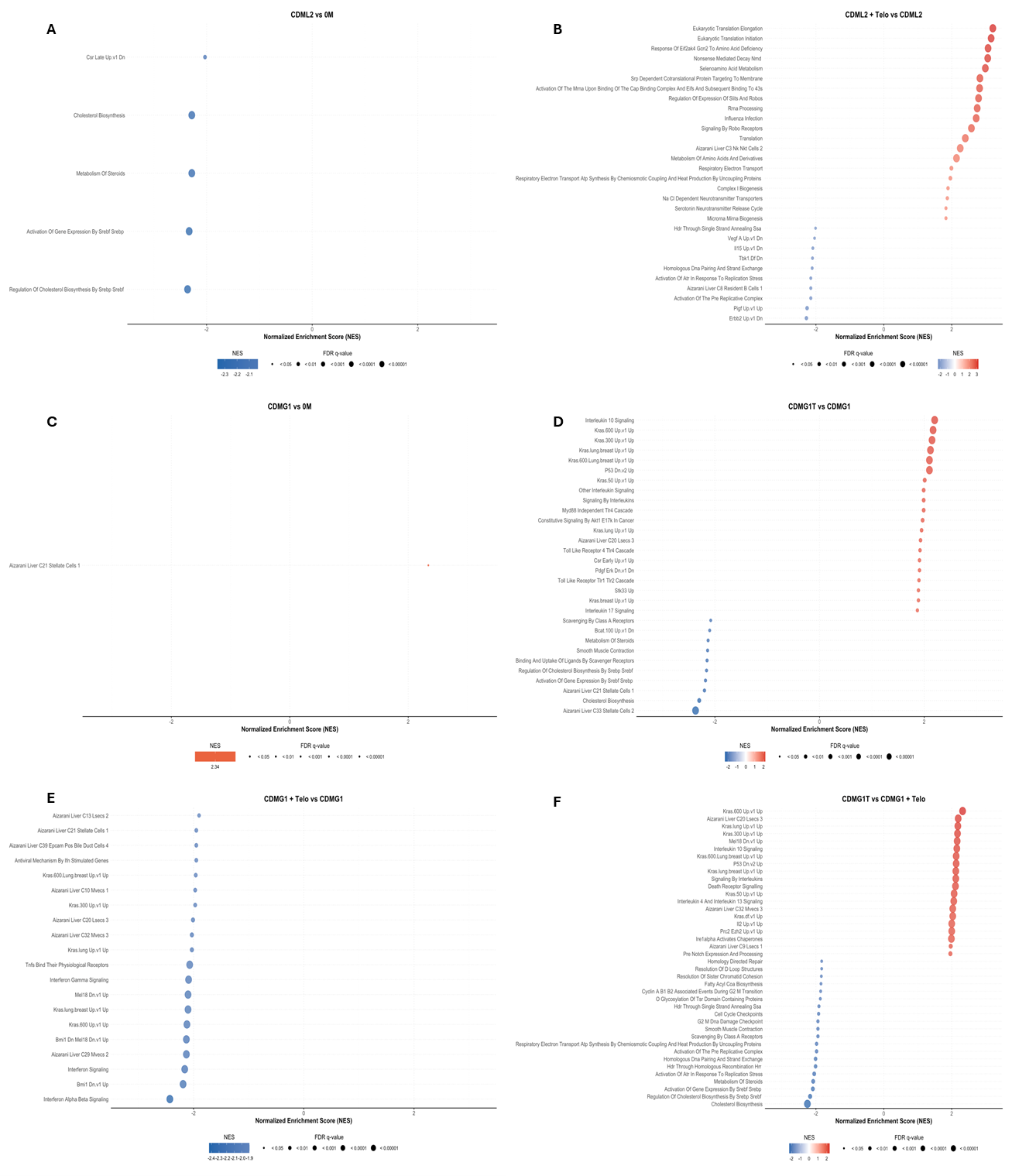
Supplementary Figure 2. GSEA data of the RNA sequencing of the cells within the paracrine *in vitro* model.**

**Supplementary Figure 2.** Gene set enrichment analysis of RNA sequencing. The top 20 positively and negatively enriched reactomes in comparisons between (A) CDML2 vs 0m, (B) CDML2 + Telo vs CDML2, (C) CDMG1 vs 0M, (D) CDMG1T vs CDMG1, (E) CDMG1 + Telo vs CDMG1 and (F) CDMG1T vs CDMG1 + Telo.
