# Supplementary material for: Telotristat ethyl affects tumour‐fibroblast crosstalk in small intestinal neuroendocrine tumours
Source: J Neuroendocrinol. 2025 Sep 25;37(11):e70094. doi: 10.1111/jne.70094 (PMC12580468; doi:10.1111/jne.70094)
Supplement: Supplementary file 2 — Table S1. Summary of demographic and clinical characteristics of patients enrolled in the study. Supporting Information Table 1. Assay‐on‐Demand primers used for RT‐qPCR. Supporting Information Table 2. Summary of primary antibodies, pre‐treatments and dilutions used for immunohistochemistry. Supporting Information Table 3. Summary of primary antibodies, hosts and dilutions used for western blot analysis. Supporting Information Table 4. Significantly changed genes between groups using RT2 profiler. [file JNE-37-e70094-s002.pdf]

Telotristat ethyl affects tumour-fibroblast crosstalk in small intestinal neuroendocrine tumours

Harry Hodgetts[1], Maria Castanho Martins[1], Luohai Chen[1,3], Andrew R Hall[1,2], Tu Vinh Luong[1,2,4], Martyn Caplin[3], and Krista Rombouts[1]

[1]Regenerative Medicine and Fibrosis Group, Institute for Liver and Digestive Health, Royal Free Hospital, University College London, London, UK,

[2]Sheila Sherlock Liver Centre, Royal Free London NHS foundation trust, London, UK

[3]Neuroendocrine Tumour Unit, ENETS Centre of Excellence, Royal Free London NHS Foundation Trust, London, UK

[4]Cellular Pathology Department, Royal Free London NHS Foundation Trust, London, UK

Correspondence should be addressed to: Harry Hodgetts, h.hodgetts@ucl.ac.uk or Krista Rombouts, k.rombouts@ucl.ac.uk

Krista Rombouts ORCID ID: 0000-0001-9440-0571

Harry Hodgetts ORCID ID: 0009-0003-9498-1961

Short Title: Telotristat ethyl on tumour-fibroblast crosstalk

Keywords: small intestine, neuroendocrine tumour, telotristat ethyl, tumour microenvironment, fibroblast.

Supplementary Information

Supplementary information 1. Multidimensional assessment of mesenteric fibrosis used to classify patients into different groups of mesenteric fibrosis severity

A multidimensional assessment of mesenteric fibrosis was used to accurately classify the mesenteric fibrosis severity, which incorporated surgical, radiological, and histological criteria, that we have previously described (Kidd*, et al.* 2013). The following components of this assessment were assessed:

i). The radiological severity of mesenteric desmoplasia was based on the scoring system originally proposed by Pantongrag-Brown *et al*, using the following categories: a. No radiological evidence of mesenteric desmoplasia (Absence of radiating strands), b. Mild desmoplasia (≤10 thin radiating strands), c. Moderate desmoplasia (>10 thin strands or <10 thick strands) and d. Severe desmoplasia (≥10 thick strands)

ii). The histological assessment of mesenteric fibrosis was based on the histological slide with the maximum amount of fibrous tissue. The histological slide was stained with a connective tissue stain (Sirius Red) and two parameters were measured:

a). The width of the thickest fibrous band surrounding the mesenteric mass. This technique was used previously by Pantongrag-Brown et al and showed a correlation with the radiological assessment of mesenteric fibrosis (Pantongrag-Brown*, et al.* 1995).

b). The Collagen Proportionate Area (CPA), which represents the percentage of collagen in the stroma surrounding the mesenteric metastatic tumour. This is a quantitative method of measuring fibrous tissue using digital image analysis and has been validated in liver cirrhosis (Laskaratos*, et al.* 2020), (Marrone*, et al.* 2018).

Optimisation/characterisation of the inter-observer variability

The cross-sectional imaging (CT/MRI scan) was assessed independently by 2 assessors (CS, JB) with good inter-observer agreement. In a small number of cases (n=3) a minor discrepancy was observed

between the two assessments and consensus was reached between the assessors to determine the radiological severity of the mesenteric desmoplasia after a final review of the imaging studies.

The histology (mesenteric mass with surrounding fibrosis) were assessed independently by 2 assessors (AH, SA) with good inter-observer variability (CPA r=0.86998 [95% CI 0.7487, 0.9347], p<0.0001; width of fibrous bands r=0.9174 [95% CI 0.8366, 0.9591], p<0.0001). In the case of minor discrepancies (<20% difference between the two measurements) the mean value of the two assessments was calculated and used for our analysis. In the small number of cases with more significant discrepancies (>20% difference between the two measurements), consensus was reached between the two assessors regarding the CPA and width of fibrous band measurements after a final review of the histology slides.

iii). A surgical (intra-operative) assessment of the extent of mesenteric fibrosis in relation to the entire small bowel mesentery was also provided using the following categories: a. No desmoplasia (No mesenteric fibrosis), b. Mild desmoplasia (Mesenteric fibrosis involving <25% of the small bowel mesentery), c. Moderate desmoplasia (Mesenteric fibrosis involving 25-50% of the small bowel mesentery) and d. Severe desmoplasia (Mesenteric fibrosis involving >50% of the small bowel mesentery). This assessment was provided by the operating surgeon (the same surgeon [OO] performed the macroscopic assessment of mesenteric desmoplasia in all the cases).

Optimal cut-off points of histological parameters (maximum width and CPA) for the prediction of clinical fibrosis

A receiver operating characteristic (ROC) curve was used to establish the optimal cut-off points of CPA and maximum width of fibrous bands that predicted with the best sensitivity and specificity the presence of clinical (radiological and/or surgical) fibrosis.

A width of fibrous band > 0.505mm and a CPA >37.6% were identified as the optimal cut-off points (Width of fibrous bands: AUC 0.751 [95% CI 0.535, 0.967], p=0.027 and CPA (AUC 0.804 [95% CI 0.634, 0.975], p=0.007).

Development of different patient groups of mesenteric fibrosis severity

A total of 34 patients were grouped in different categories of mesenteric fibrosis severity:

1). Non-fibrotic group: No evidence of mesenteric fibrosis (n=3)

2). Minimally fibrotic group: Only histological (but no clinical evidence of) fibrosis (n=6). In this group of patients, a mesenteric metastasis was present and surrounded by a small amount of fibrosis detected only histologically.

3). Mildly and severely fibrotic groups: These patients had evidence of clinical (macroscopic) fibrosis (n=25). The group of patients with clinical fibrosis was further sub-divided into 2 smaller subgroups: A group of patients with mild fibrosis (n=14) and another group with severe fibrosis (n=11). A scoring system was developed to group patients in different categories of clinical fibrosis severity. This scoring system (**Table**) incorporated surgical (macroscopic), radiological and histological parameters.

Table. Scoring system used to assess the severity of mesenteric fibrosis. This system is not validated, but incorporates surgical, radiological and histological measurements, therefore allowing for a more objective, multidimensional assessment of the severity of mesenteric fibrosis (Kidd*, et al.* 2013). CPA: Collagen Proportionate Area.

**Surgical evidence of fibrosis**	**Radiological evidence of fibrosis**	**Histological evidence of fibrosis**
0: None	0: None	0: None

1: <25% of small bowel mesentery	1: ≤10 thin strands	1: Yes, but CPA<37.6% AND width of fibrous band <0.505 mm
2: 25-50% of small bowel mesentery	2: >10 thin and <10 thick strands	2: Yes, CPA>37.6% OR width of fibrous band >0.505 mm
3: >50% of small bowel mesentery	3: ≥10 thick strands	3: Yes, CPA>37.6% AND max width >0.505mm

A total score of 6 was arbitrarily chosen as a cut-off point to allow a fairly equal distribution of patients in the two subgroups of clinical fibrosis (mild <6, severe ≥6).

Interestingly, the non-fibrotic group was characterised by the absence of a mesenteric mass, while all patients with a mesenteric metastasis had evidence of fibrosis development, and the extent of the desmoplastic reaction varied significantly, from minimal (detected only histologically) to more advanced.

Using this methodology, patients were grouped into 4 distinct categories with graded severity of mesenteric fibrosis, i.e. patients with no mesenteric fibrosis (n=3), minimal fibrosis (microscopic fibrotic capsule around the mesenteric lymph node) (n=6), mild (n=14) and severe mesenteric fibrosis (n=11).

Table S1. Summary of demographic and clinical characteristics of patients enrolled in the study

**Demographic and clinical characteristics**	**Patients with midgut NETs who underwent surgery (n=34) n (%)**
**Age (mean±SD)**	61±13
**Sex**	

**Male**<br><br>**Female**	23 (68%)<br><br>11 (32%)
**Grade**<br><br>**1**<br><br>**2**	 <br>21 (62%)<br><br>13 (38%)
**Extent of disease**<br><br>**Localised**<br><br>**Locoregional**<br><br>**Metastatic**	 <br>3 (9%)<br><br>9 (26%)<br><br>22 (65%)
**Mesenteric mass**	31 (91%)
**Liver metastases**	17 (50%)
**Distant extrahepatic metastases**	10 (29%)
**Mesenteric fibrosis**	31 (91%)
**Medical therapy**<br><br>**Octreotide LAR**<br><br>**Lanreotide Autogel**	 <br>10 (29%)<br><br>8 (24%)
**Surgical therapy**<br><br>**Small bowel resection**<br><br>**Right hemicolectomy (R0)**	 <br>1 (3%)<br><br>24 (71%)<br><br>9 (26%)

**Right hemicolectomy (R1)**	

Supplementary table 1. Assay-on-Demand primers used for RT-qPCR.

Gene	Assays-on-demand Primer
COL6A2	Hs00365167
ITGA3	Hs01076879
LAMA5	Hs00966585
COL12A1	Hs00189184
GAPDH	Hs02786624
ITGA9	Hs00979865
NCSTN	Hs00299716
VCL	Hs00247826
DCN	Hs00370384
EGF-R	Hs01076078
PLAT	Hs00938315
CCL3	Hs00234142
NOTCH1	Hs01062014

Supplementary table 2. Summary of primary antibodies, pre-treatments and dilutions used for immunohistochemistry.

Primary antibody	Host	Company	Product code	Pre-treatment	Dilution
CDH1	Rabbit pAb	Novus Biologicals	NBP1-84588	Microwave in pH6.0 citrate buffer for 20mins	1:250
P4HB	Rabbit pAb	Novus Biologicals	NBP1-84051	Microwave in pH6.0 citrate buffer for 20mins	1:4000
TIMP1	Rabbit pAb	Abcam	Ab231485	Heat at pressure for 6mins in pH 6.0 citrate buffer for 20mins	1:200

Collagen type IV	Mouse mAb	Dako	M0785	Trypsin/chymotrypsin digestion, pH7.6 at 37°C for 30 mins	1:75
ADAM12	Rabbit pAb	Abcam	Ab223745	Microwave in pH6.0 citrate buffer for 20mins	1:400
Matriptase-2 (TMPRSS6)	Rabbit pAb	Novus Biologicals	NBP1-57098	Heat at pressure for 6mins in pH 6.0 citrate buffer for 20mins	1:250

Supplementary table 3. Summary of primary antibodies, hosts and dilutions used for western blot analysis.

Primary Antibody	Host	Company, Cat. No	Blocking Buffer	Primary Dilution	Secondary Dilution
BiP	Rabbit pAb	Cell Signaling Technology, 3177	5% BSA	1:1000	1:2000
pAKT	Rabbit pAb	Cell Signaling Technology, 4060	5% BSA	1:2000	1:2000
AKT	Rabbit pAb	Cell Signaling Technology, 4685	5% BSA	1:1000	1:2000
pERK	Rabbit pAb	Cell Signaling Technology, 4370	5% BSA	1:2000	1:2000
ERK	Rabbit pAb	Cell Signaling Technology, 4695	5% BSA	1:1000	1:2000
aSMA	Mouse pAb	Sigma, A2547	5% BSA	1:10,000	1:10,000
b-catenin	Rabbit pAb	Cell Signaling Technology, 8480	5% Milk	1:1000	1:2000

Supplementary table 4. Significantly changed genes between groups using RT2 Profiler

Cell type	Conditions	Gene symbol	Fold change	P value
GOT1	CM vs. 0M	*CA9*	2.41	0.037902
		SNAI2	3.01	0.000372
		TBX2	2.34	0.010145
	CdML2 vs. 0M	*GADD45G*	1.54	0.000055
	CdML2T vs. 0M	No changed genes found		
	CdML2+Telo vs. 0M	*CA9*	4.73	0.002304
	CdML2T vs. CdML2	*HMOX1*	2.34	0.043715
		FOXC2	-1.53	0.001982
	CdML2+Telo vs. CdML2	No changed genes found		
LX2	CM vs. 0M	*ACTA2*	4.92	0.000045
		EDN1	4.08	0.000076

		EGF	3.68	0.000243
		ITGB3	2.04	0.00215
		LOX	2.4	0.000064
		SERPINE1	2.32	0.000047
		SMAD6	3.52	0.000104
		SMAD7	4.83	0.000141
		TGFB2	2	0.000881
		THBS1	1.73	0.000262
		THBS2	2.54	0.000117
		ACTB	1.69	0.00221
		IL1A	-1.86	0.005277
		IL1B	-3.85	0.000886
		IL5	-3.21	0.046444
		ITGA1	-1.9	0.000425
		ITGA2	-3.51	0.005439
		ITGA3	-1.58	0.001379
		ITGB8	-1.82	0.002222
		MMP9	-2.65	0.00006
		PDGFB	-1.87	0.006823
		PLAT	-1.75	0.022841
		PLAU	-2.76	0.012256
		SERPINA1	-1.69	0.000051
		VEGFA	-1.54	0.000865
	CdMG1 vs. 0M	*CCL3*	2.24	0.049356
		PLAT	1.99	0.007445
	CdMG1T vs. 0M	*PLAT*	2.05	0.020348
		COL3A1	-1.63	0.003117
		TGFB2	-1.68	0.005672
	CdMG1+Telo vs. 0M	*ITGA2*	-1.74	0.032651
		MMP3	-1.79	0.010876
		MMP9	-1.53	0.001673
		SERPINA1	-1.63	0.005646
	CdMG1T vs. CdMG1	*TGFB2*	-1.51	0.009748
	CdMG1+Telo vs. CdMG1	*CCL3*	-2.4	0.047573
		PDGFB	-1.53	0.004535
		PLAT	-1.71	0.021959
		SNAI1	-1.54	0.019789

Abbreviations: 0M, medium with 0.5% FBS; CM, complete medium with 10% FBS; CdML2, conditioned medium rom LX2 cells; CdML2T, conditioned medium from telotristat ethyl treated LX2 cells; CdML2+Telo, conditioned medium from LX2 cells supplemented with telotristat ethyl; CdMG1, conditioned medium from GOT1 cells; CdMG1T, conditioned medium from telotristat ethyl treated GOT1 cells; CdMG1+Telo, conditioned medium from GOT1 cells supplemented with telotristat ethyl.

References

Kidd M, Schimmack S, Lawrence B, Alaimo D & Modlin IM 2013 EGFR/TGFalpha and TGFbeta/CTGF Signaling in Neuroendocrine Neoplasia: Theoretical Therapeutic Targets. *Neuroendocrinology* 97 35-44.

Laskaratos FM, Mandair D, Hall A, Alexander S, von Stempel C, Bretherton J, Luong T, Watkins J, Ogunbiyi O, Rombouts K, et al. 2020 Clinicopathological correlations of mesenteric fibrosis and evaluation of a novel biomarker for fibrosis detection in small bowel neuroendocrine neoplasms. *Endocrine* 67 718-726.

Marrone G, De Chiara F, Bottcher K, Levi A, Dhar D, Longato L, Mazza G, Zhang Z, Marrali M, Fernandez-Iglesias A, et al. 2018 The adenosine monophosphate-activated protein kinase-vacuolar adenosine triphosphatase-pH axis: A key regulator of the profibrogenic phenotype of human hepatic stellate cells. *Hepatology* 68 1140-1153.

Pantongrag-Brown L, Buetow PC, Carr NJ, Lichtenstein JE & Buck JL 1995 Calcification and fibrosis in mesenteric carcinoid tumor: CT findings and pathologic correlation. *AJR Am J Roentgenol* 164 387-391.
